# A longitudinal study on the performance of in vivo methods to determine the osteochondrotic status of young pigs

**DOI:** 10.1186/s12917-016-0682-z

**Published:** 2016-03-24

**Authors:** Christian P. Bertholle, Ellen Meijer, Willem Back, Arjan Stegeman, P. René van Weeren, Arie van Nes

**Affiliations:** Department of Farm Animal Health, Faculty of Veterinary Medicine, Utrecht University, Yalelaan 7, NL-3584 CL Utrecht, The Netherlands; Department of Equine Sciences, Faculty of Veterinary Medicine, Utrecht University, Yalelaan 112-114, NL-3584 CM Utrecht, The Netherlands; Department of Surgery and Anaesthesia of Domestic Animals, Ghent University, 17 Salisburylaan 133, B-9820 Merelbeke, Belgium

**Keywords:** Pig health, Osteochondrosis, Lameness, Gait analysis, Radiography, Histology, Early detection

## Abstract

**Background:**

In today’s porcine industry, lameness has a major welfare and economic impact, and is often caused by osteochondrosis (OC). The etiological factors of the disease have been studied in depth, however, to this day, little is known about the natural course of the disorder and how it can be detected at an early stage in pigs. The aim of this pilot study was to assess the potential of three non-invasive techniques for the detection and monitoring of early OC processes in piglets. A group of weaned piglets (*n* = 19) were examined longitudinally using radiographs, a visual lameness scoring scheme and a quantitative pressure-mat based locomotion analysis system to detect OC in the humeroradial, femoropatellar and tarsocrural joints. At several time points, a selection of animals was euthanized for post-mortem examinations, including histology, which was the gold standard.

**Results:**

In this study, clear signs of subclinical signs of OC were observed, however, we were unsuccessful in producing clinical OC. Lesions were observed to be commonly bilaterally symmetric in the joints examined in 80 % of cases. The radiographic examinations showed a clear correlation with the gold standard, particularly when subclinical lesions were of a high histological score. Moreover, radiography was also able to detect the early repair processes, which appeared to take place at least until 14 weeks of age. Both visual scoring and pressure mat analyses showed good intra-assay reproducibility, with the pressure mat showing intra-class correlation values between 0.44 and 0.6 and the inter-observer agreement of visual scoring method was between 88 and 96 %, however their correlation to OC lesions detected by histology was very weak, with only 2 out of 12 traits for the visual scoring method showing significant and biologically logical relations to a specific joint having histological OC lesions. For the pressure mat, only a maximum of 5 associations for specific joints with histological OC lesions were found out of a possible 8.

**Conclusion:**

All tested in-vivo methods showed good reproducibility. Radiography was the most reliable technique to detect and monitor longitudinally the earliest signs of OC in these piglets. It also demonstrated that the “Point of No Return” (PNR) of the disease, when repair processes end, might be later than anticipated, after 13 weeks of age. All in all, our study shows that the timing of the use of these in-vivo methods is critical to detect and monitor OC, especially in the early phases of the disease. It also shows the difficulty in producing OC regardless of the optimization of the experimental settings in relation to the etiological factors known to induce OC.

**Electronic supplementary material:**

The online version of this article (doi:10.1186/s12917-016-0682-z) contains supplementary material, which is available to authorized users.

## Background

Osteochondrosis has often been reported to be the main cause of lameness in pigs [1–3] and is still currently causing substantial economic losses and alarming welfare concerns for both sows and slaughter pigs [4]. It is estimated that 80 % of the pigs in today’s porcine industry show superficial to mild signs of OC [1]. OC has been defined as a multi-focal disturbance in the endochondral ossification process that occurs during skeletal growth, leading to lesions in both the articular and physeal cartilage [1, 5]. Many advances have been achieved in the understanding of the disease process, for example with respect to the identification of etiological factors. However, little is known still on the natural course of the disease and especially on the early stages [5]. Nevertheless, the fact that the molecular and cellular structure of epiphyseal cartilage and that the mechanism of disruption of the endochondral ossification process are similar in different species, lead us to believe that these early stages could also be comparable in the pig [6–8]. This means that recent findings in equine medicine on OC, where it is suggested now that there is a dynamism of the interplay process between lesion initiation and early repair, could be extrapolated to porcine OC [9, 10]. Additionally, the existence of a Point of No Return (PNR) has also been discovered in the horse. In the pig, this PNR is anticipated to be before 13 weeks of age, after which lesions can no longer be repaired, as vascularization of the cartilage tissue has disappeared [11, 12]. The understanding of OC has for a long time principally relied on data that was acquired by either radiography or post-mortem analysis [13], however more recent techniques have also shown promising results, such as micro-CT [14, 15]. In the pig, good correlations have been reported between radiographic results and post-mortem macroscopic and microscopic data, although this was in animals showing more the later clinical stages of the disease [13], than the early stages or subclinical signs of OC. In spite of the valuable performance of radiography, there is a need in the field to have other effective in-vivo detection methods able to detect the early signs of OC in a higher through-put way. In other species, such as the horse, the dog and cattle, the development of locomotion in relation to leg and claw disorders has been studied successfully using modern gait analysis techniques [16–18]. In this pilot study, we investigated the frequency of OC in our pigs and per joint. We studied also the bilateral symmetry of OC, and the efficacy and suitability of three non- invasive techniques to detect early signs of OC, by comparing them to the gold standard (histological examination). These in-vivo techniques included the visual scoring of the conformation and gait of the animals, the use of a pressure mat to quantify gait and radiographic screening.

## Results

### Health-prevalence of OC-bilateral symmetrical aspect

**Fig. 1:**
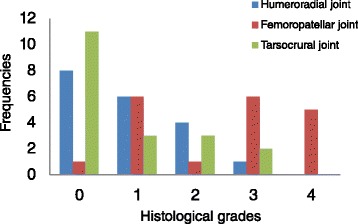
Histology scores for *n* = 19 pigs (using both left and right sides of the joint were combined and the overall worst score from all investigated locations within the joints)

Average daily weight gain of the 19 pigs was 253 g/day (+/− 246 g) and average body weight was 6.5 kg (stdev. +/− 0.64) and 34.2 kg (stdev. +/− 0.79), at 4 and 14 weeks of age respectively. At necropsy, no macroscopic OC lesions were detected in any of the joints in any of the pigs. At histology, most of the lesions were observed in the femoropatellar joint, which featured the most severe lesions (Fig. 1). The OC status appeared to be highly symmetrical in left and right joints for all three joints with an identical histological score (H score) or an H score differing only by one unit in over 80 % of cases (Table 1).

**Table 1:** Left/right comparison of OC histological status (*n* = 19)

	Humeroradial (*n* = 32^a^)	Femoropatellar (*n* = 39^a^)	Tarsocrural (*n* = 25^a^)
Bilateral lesions (both scores >1)	9.4 %	33.4 %	8.0 %
Bilateral no lesions (both scores 0)	56.3 %	23.1 %	68.0 %
1 side score 0, the other score >1	31.3 %	25.6 %	12.0 %
1 side score 0, the other score >2	3.1 %	18.0 %	12.0 %

^a^Only the outcomes where left and right histology scores were available for each location

### In-vivo versus post-mortem gold standards (Radiographic versus Histological examination)

**Fig. 2:**
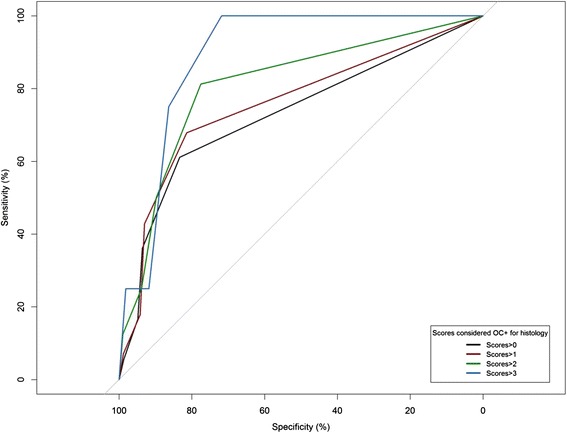
ROC curves showing the difference in sensitivity and specificity of radiography versus the gold standard, histology using various thresholds

The comparison between histology and radiography was done using the data from both techniques from the 10 radiographed pigs at the last time point. Fig. 2 shows a receiver operating characteristic (ROC) curve describing the sensitivity/specificity of radiographic results for all joints with different cut-offs of histology, considered as the gold standard, Table 2 shows the statistical difference in sensitivity and specificity between the radiographs and histology. Results show that the sensitivity increases and the specificity decreases, as the threshold setting for histology increases.

**Table 2:** Description of the sensitivity and specificity of radiography for the detection of OC as defined by the gold standard, histology (*n* = 10)

	Specificity (%)	Sensitivity (%)	AUC	Confidence interval
Roc 1 (Both H&R scores >0)	83.3	61.1	72.3	63.61–82.22 %
Roc 2 (Both H&R scores >1)	81.4	67.8	75.3	65.66–85.79 %
Roc 3 (Both H&R scores >2)	77.5	81.2	80.9	69.49–92.24 %
Roc 4 (Both H&R scores >3)	71.8	100.0	89.1	79.9 %–98.2 %

*R scores* radiographic scores, *H scores* histological scores, *AUC* area under the curve, *ROC*

When examining the different areas under the curve (AUC), which measure the general accuracy of the test, for each threshold setting, the range is chronological from 72.3 % (for H and R scores >1) to 89.1 % (for scores above 3). This demonstrates a good overall specificity and sensitivity of radiographs compared to the gold standard, which appears to increase as the histological lesions become more severe. Concerning the correct identification of negative joints for OC, radiography has a good specificity from R score 0 up to R score 2, but is accompanied by a low sensitivity.

**Fig. 3:**
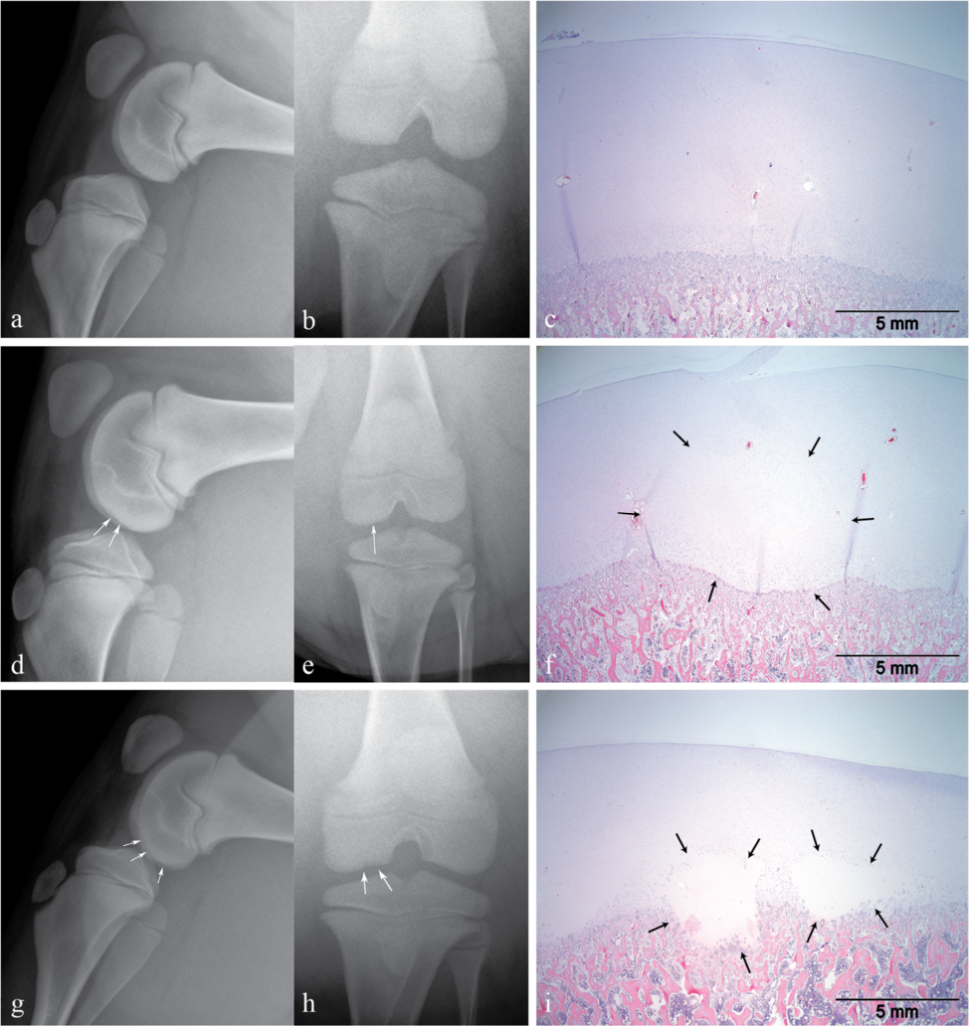
**a**–**i**. Typical images of the histological grades with corresponding radiographic findings. Photo pairs **a**, **b**, **d**, **e** and **g**, **h** are radiographic images taken of 3 different respective pigs at 14 weeks of age. Pictures C, F and I are the corresponding histological slides in chronological order to the radiographic paired images. All stained with hematoxylin and eosin. All pictures depict assessments of the femoropatellar joint with G/H/I being from the left limb only. **a**–**c** represent the lateral side and **d**–**f** and **g**–**i** represent the medial side of the distal femoral condyle. All three pairs represent animals with no OC lesions present (**a**–**c**), with mild OC lesions (*R* = 2 and *H* = 3, **d**–**f**) and with severe OC lesions (*R* = 4 and *H* = 4, **g**–**i**)

Above R score 2 (R scores 3–4) the opposite occurs: the sensitivity dramatically increases up to 81.2 % compared to 67.8 % (for R score 2), and specificity decreases, demonstrating that radiography identifies positive histological joints better from R score 3 onwards (Table 2). Overall, from these results we can establish that there is a good relation between histology and radiography and additionally both sensitivity and specificity vary with the cut-off. Figure 3 shows typical examples of histological lesions with the corresponding radiographs.

### Longitudinal radiographic monitoring

Our results showed that lesions remained stable (80–95 % of lesions) except for the femoropatellar joint, where progression, regression and even resolution of lesions were apparent (Table 3). Resolution occurred in all joints after the hypothetical timing of the PNR (7 weeks of age < PNR < 13 weeks of age).

**Table 3:** Frequencies of lesion development detected radiographically at 7, 11 and 14 weeks of age (*n* = 20, for 10 pigs and 2 sides for each joint)

	Grade	Lesions present	Stationary	Progression	Regression	Resolution
7–11 weeks						
Humeroradial	0	13	13	0	0	0
	1–4	7	4	0	0	3
Femoropatellar	0	11	3	0	0	0
	1–4	9	2	11	0	4
Tarsocrural	0	18	17	0	0	0
	1–4	2	0	1	0	2
11–14 weeks						
Humeroradial	0	16	14	0	0	0
	1–4	4	2	2	0	2
Femoropatellar	0	7	3	0	0	0
	1–4	13	7	5	2	3
Tarsocrural	0	19	19	0	0	0
	1–4	1	0	0	0	1

### Visual scoring reproducibility

Inter-observer agreement was good to excellent (88–96 %) for all scored parameters but one: (sickled-buckled fore, Table 4).

**Table 4:** Percentages of the frequencies of all visual scoring categories scored (VS scores) by both raters for all pigs measured at all time points

Traits	Agreement (%)	Disagreement (%)	Missing scores (%)
O or X shape fore legs	95	4	1
Sickled or Buckled fore legs	46	54	0
Steep/Low angled Pasterns fore	94	6	0
Claw size fore	99	0	1
O or X shape hind legs	88	11	1
Straight or Sickled hind legs	94	5	1
Steep/Low angled Pasterns hind	91	8	1
Claw size hind	99	0	1
Ham	91	8	1
Gait pattern	93	6	1
Twisting hocks	99	0	1
Swaying hind	95	4	1

### Pressure mat reproducibility

Table 5 gives intra-class correlation (ICC) data on the pressure mat kinetic factors showing overall excellent reproducibility (ICC values 0.44 < x < 0.6).

**Table 5:** Intra-class correlation data for the pressure mat parameters

	Average	Average	Average	Average
	FMAX	VI	CA	Pmax
ICC(1)	0.58	0.60	0.56	0.44
F-Test	5.51	5.75	4.98	3.50
P value	<0.05	<0.05	<0.05	<0.05

### Comparison of visual scoring and histology

When considering visual scoring and histology, 12 pigs were selected on the basis that they were the only ones showing the highest number of results present for both techniques, in a total of 9 joint locations (2 in the humeroradial joint, 4 in the femoropatellar joint and 3 in the tarsocrural joint). This comparison showed that the hind pasterns was the only element that showed a significant relationship at joint level, once the Benjamini and Hochberg false discovery rate correction was applied to all the Spearman correlation test results (Table 6).

**Table 6:** Associations between visual scoring and histology (9 joint locations - *n* = 12)

Visual scoring element	Animal or Joint level	Spearman’s rank correlation test	Benjamini and Hochberg correction
Pasterns hind	Femoropatellar	−.786 (*P* = 0.00)	0.01

### Comparison of pressure mat and histology

Using the same set of pigs as for the visual scoring/histology comparison, four comparisons between the ASIs (Asymmetry Indexes) and histology were tested. The first two were based on the ASIs and the transformed H scores. Only Fore/Hind ASIs showed relations with Animal and Femoropatellar levels (Table 7).

**Table 7:** Associations between pressure mat and histology data (9 joint locations - *n* = 12)

Pressure mat ASIs of kinetic factors		Histology OC score level (Joint or animal level)	Spearman’s test	Benjamini and Hochberg correction
Fore/Hind ASIs	ASI CA Right	Animal	−0.772 (*p* = 0.00)	0.05
	ASI Pmax Right	Femoropatellar	0.822 (*p* = 0.00)	0.03
	ASI Pmax Left	Femoropatellar	0.751 (*p* = 0.00)	0.05

Thirdly, an “ASI animal level score” (sum of all ASI absolute values) was compared to the H score on animal level, but no associations were found (results not shown). Finally, a direct comparison between ASIs of severe histologically OC Positive joints (H scores >3) and those of OC negative joints was tested. Only ASI Pmax fore (Right/Left setting) showed a significant link with the presence and absence of severe histological OC lesions (results not shown).

## Discussion

### Frequency, severity of and bilateral symmetrical aspect of OC lesions

In the present study, 64.9 % of the animals showed microscopic signs of OC, with 24.6 % having histologically severe lesions (H scores > 3; Fig. 1). The femoropatellar joint had the highest incidence of lesions and the highest number of high grade lesions. Nevertheless, in clinical terms, only 7 % of animals lesions of a high grade radiographically (R scores > 3) and at necropsy, no lesions were macroscopically visible in any of the pigs. We were therefore unexpectedly unsuccessful in creating clinically OC, despite using environmental conditions (slippery flooring) and nutritional regimens (high protein diets fed ad-libitum) designed to promote the development of the disorder [5]. The advantage of this situation was that we were able to assess our non-invasive methods under challenging conditions. Few studies have looked at the the frequency of OC in pigs on a longitudinal scale and in the early subclinical phases of OC. Some studies reported frequencies from 41.4 % using macroscopic evaluation to 78 % using microscopy [18]. There are also large differences with respect to the frequency per joint, with some studies reporting the tarsocrural joint as the joint to be most commonly affected [19], while others mention the medial condyles of the femur and humerus [13]. The techniques used to detect OC were similar in nature, thus these differences are most likely linked to the multifactorial aspect of this diseases, which can affect one population of identical genetic pigs differently if they were exposed to different environmental conditions for example. Regarding the bilateral symmetrical effect, the humeroradial joint was the most consistent in showing this effect in our study (Table 1). OC is generally seen as a multifocal disease with a relatively strong bilateral occurrence of lesions [5, 20, 21]. This finding has even prompted some studies to analyse solely one side of the animal [13]. Our results confirm these previous findings.

### In-vivo radiography as a diagnostic tool for OC detection compared to histology

Unsurprisingly, the different AUC results measuring the accuracy of radiographs versus histology showed that radiography performed best for detecting the most severe histological lesions (Table 2). Additionally, in terms of specificity, radiography showed that all histological lesions from H score 2, 1 and 0 were identified as OC negative. This was expected, as these lesions are mostly mild subclinical ones and would be therefore challenging to visualize with a clinical tool, such as radiography. All things considered, from these results, we can conclude that the most reliable threshold for radiography, to identify correctly the OC status of these joints, is situated around H score 3, when lesions are at high score histologically. This observation is important, as it demonstrates the ability of radiography to detect only the more severe subclinical OC lesions in pigs reliably. Although radiography is considered currently as the standard imaging method to diagnose clinical OC [22], and most studies have investigated OC in other species [7, 21–23], not many have focused on pigs [24, 25]. Other studies have also shown significant correlations between both methods, which were joint dependent [13, 26], however these studies were performed on older pigs and foals. Our results show a similar relevant correlation between both methods. In terms of monitoring, the results showed that lesions occur already at 7 weeks of age, and are mostly of a low grade nature for radiological standards (R scores <3). Although OC lesions progressed, as expected, between 11 to 14 weeks of age, mostly in the humeroradial and femoropatellar joints, simultaneous repair processes were also taking place. This means that the PNR, which was anticipated to be before 13 weeks of age, is probably situated somewhat later in time. In horses, OC is known to be a very dynamic disease in which lesions appear, but then may, to a large extent, resolve spontaneously afterwards as a repair process starts immediately after occurrence of a lesion [27]. For the pig, we can conclude that a similar dynamic picture emerges, with differences per joint. Unexpectedly, there does not seem be a straightforward relation with the time window until 7–13 weeks of age, when the growth cartilage is still vascularized [11, 12]. The current paradigm is that OC is very likely caused by a failure of the vascular supply of the growth cartilage through cartilage canals in specific focal areas, which then leads to small necrotic areas that are not yet visible radiographically and are hence called osteochondrosis latens. If not resolved, these lesions may become larger and of clinical relevance, at which stage the term osteochondrosis manifesta is used [27]. Whether or not OC latens lesions will turn into OC manifesta lesions depends on both the initial size of the lesions and the repair capacity of the cartilage, which decreases rapidly with time, as the animal gets older [28]. This latter aspect is influenced by the changes in vascularization of the epiphyseal growth cartilage due to the ongoing process of endochondral ossification that will ultimately lead to the mature state in which there is only avascular articular cartilage left.

### Visual scoring and pressure mat reproducibility and comparison with histology

In this study, visual scoring of lameness and the pressure mat showed both a good reproducibility within session measurements (Tables 4 and 5). Only one relationship was found between the hind pasterns and the femoropatellar joint. In the literature, visual assessment as a potential indication of OC has only been compared with macroscopic joint assessments thus far [19, 29]. Some reports claim no relationship [30, 31], or weak associations [13], whereas others claim to have found positive associations when lesions were severe [32], or even strong associations between leg weakness and OC macroscopic scores, at joint level only [33], or animal and joint level [29]. Our results hence seem to reflect the current contradictory position in the literature regarding this relation. Until recently, quantifying lameness was usually performed by a force plate system, measuring the ground reaction forces. However, our more recent pressure mat system is able to provide information on the pressure distribution pattern of the foot or claw, and has already been used successfully in recent studies in horses, cattle, dogs and pigs [16–18, 34], however never in this study format for detecting OC. For the pressure mat, very few associations with histological data were established in this study, mostly with hind limb and animal level parameters. Other more direct comparisons between the two methods were unsuccessful. The reason for this could be the low number of pigs having clinical OC, and the presence of only sub clinical OC. This would be the reason why conformational changes, in terms of lameness, are not occurring due to OC, or that if there are conformational changes taking place, that they are actually occurring but they are not linked to OC. This makes it difficult to appreciate the full potential of the pressure mat in detecting the earliest signs of OC in-vivo, as intended in this study, and requires more testing to be performed on larger populations with more clinical OC present.

## Conclusions

Whereas the frequency of clinical OC lesions in this study was much lower than expected, histological prevalence of OC lesions was relatively high, especially in the femoropatellar joint, providing a good opportunity to assess the potential value of the in-vivo measuring systems under scrutiny for the detection of subclinical porcine OC. Moreover, as in other studies, the strong bilateral character of OC was confirmed. From the positive correlations found between radiological and histological findings in all joints and especially in the femoropatellar joint, we can conclude that in-vivo radiography performs well in terms of diagnosing and monitoring OC only at high radiographic scores and works safely in pigs at a young age. Lesions were already radiographically detected at 7 weeks of age and progression and regression/resolution of lesions continued until 14 weeks of age, which suggests that the PNR may occur later in time, than previously expected. Both visual scoring of lameness and pressure mat measurements appeared to be repeatable and well feasible in a logistical sense, but their relationship to the gold standard was weak and they are not apt to detect subclinical OC. Nevertheless, further development of the pressure mat approach seems to show potential for high-throughput assessments of joint disorders in pigs, even at a very young age and warrants further exploration in more heavily affected populations and over a longer timescale.

## Methods

### Animals and experimental set-up

Nineteen “Topigs 20” piglets at weaning age (4 weeks of age, Van Beek BV, Lelystad, NL) were used for this study. Male-female numbers were close to a 50/50 ratio with 10 males and 9 females to have a good gender distribution. The animals were housed in three groups in relatively equal size pens (153 cm × 256 cm) equipped with toys and smooth wooden plates covering half of the floor to increase slipperiness and the chance of developing OC [20]. An 18 h light-dark cycle was used and stall temperature was set at 24 °C. Pigs were fed ad libitum with a customised diet with a high concentration of proteins (20 % feed) including a high volume of lysine (14.0 g/kg feed) and without cartilage residues (Research Diet Services B.V., Wijk bij Duurstede, NL) and had constant access to water. The pigs were housed for 1 week on arrival for adaptation and training purposes, and were 14 weeks old at the end of the study. The study was divided into four periods of three weeks, separated by weekly in-vivo measuring time-points. Every week, all pigs were weighed using a standard scale (Schippers BV, Bladel, NL). At each period, randomly selected pigs were euthanized and the humeroradial, femoropatellar and tarsocrural joints were examined macroscopically and histologically at specific locations for the presence of OC lesions. All joints were examined specifically at specific sites known to develop osteochondrotic lesions (24 total). The list of sites is based on a previous study for macroscopic evaluations in pigs that originated from horses [19]. Three pigs were euthanized at the first three time points and ten at the last one. The ten pigs, of the last time point, were the only ones to be radiographed. Radiographic monitoring of these pigs took place at the last 3 time points (Additional file 1).

### Visual scoring

The method used to score the pigs was derived from a scoring system previously devised by Van Steenbergen [35] and used in a previous study on OC [29]. Briefly, nine anatomical and three mobility categories were scored each with two opposing traits. In this study, due to the small number and young age of the pigs, we regrouped the 1–9 category scale system into a three-point scale (Additional file 2).

### Pressure mat analysis

**Fig. 4:**
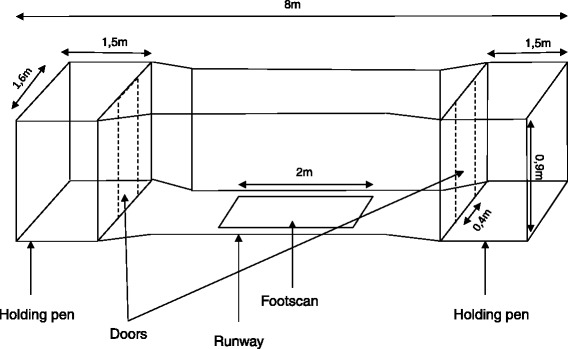
Schematic drawing of the custom-built runway with a pressure mat for quantitative gait assessment

The claw pressures were measured using a Footscan 3D 2 m-system (RsScan International, Olen, Belgium) containing 16 384 force pressure sensors operating at a frequency of 126 Hz. The mat was protected by a 0.5 mm thick mat (shore value 65° ± 5) and was set into a “runway” system so the pigs could “strike” the mat precisely (Fig. 4). Pigs were trained in the first week to perform “successful runs”, in which they trotted smoothly, in straight line and with their heads staying horizontal. To avoid any influence of velocity and other interfering parameters (e.g. growth) on the results, asymmetry indexes (ASI’s) of the Peak Vertical Force (Fmax in N), Peak Vertical Pressure (Pmax in N/cm^2^), Contact Area (CA in cm^2^) and Vertical Impulse (VI in Ns) were used as outcome parameters, as previously described in Meijer et al. [31]. The ASIs were calculated using the following formula: [(Left parameter − Right parameter)/0.5X (Left parameter + Right parameter)]X100.

### Radiographical analysis

Radiographs were taken of the ten pigs that were euthanized at the end of the study, at 8, 11 and 14 weeks of age. The images were taken with a two-view setting for all three joints on both sides (Left and Right): a lateromedial view and cranial/dorsal view, using a Philips Optimus full radiography digital system with flat panel detectors (FPDs). Settings for the contrast (mA) and density (kVp) were for pigs at 8 weeks of age: 55 kV and 5.5mAs for all joints (no grid used) using medial/lateral and cranial caudal views. The settings for the larger pigs (11 weeks and 14 weeks old) medial/lateral views (no grid) were used with 60 kV and 8mAs settings for the humeroradial and femoropatellar joints. These 2 joints had also a caudal cranial view taken (with grid) at 60–63 kV and 10–12.5mAs. For the tarsocrural joint we used a plantar/dorsal and medial/lateral view (no grid) with a 55–60 kV and 5-10mAs setting. The variations in the settings are due to the fact settings had to be adapted due to the growth of the animals during the study. Prior to radiography, the pigs were sedated using Stresnil (azaperone 40 mg/ml-Janssen Animal Health Benelux) intramuscularly, in a dose of 1 ml/10 kg. X-rays were assessed independently by 2 veterinary radiologists, using a 0–4 scale adapted from a grading system previously used in horses [9]. To allow a better comparison between radiography and histology, the joints were imaged in such a way that they would encompass the same locations that were assessed with histology. The grading is detailed in Table 8.

**Table 8:** Radiography grading (from Dik et al. [9])

Grade	Classification	Bone contour	Subchondral bone texture	Fragment
0	Normal	Rounded	Diffuse density	Absent
1	Minimal	Smoothly flattened	Obscure lucency	Absent
2	Mild	Irregularly flattened	Obvious, ill-bordered local lucency	Absent
3	Moderate	Small, rounded/irregular concavity	Obvious, well-bordered local lucency	Small fragment(s)
4	Severe	Large, rounded/irregular concavity	Obvious, well-defined extensive lucency	Large fragment(s)

### Macroscopic grading of joints

At 5, 8 and 11 weeks of age, three pigs were euthanized at each time point and at 14 weeks of age for the remaining ten pigs were euthanized. Euthanasia was carried out using pentobarbital (200 mg/kg) injected intracardially (Euthanimal, Alfasan, Woerden, NL) after they had been previously sedated with Stresnil (azaperone 40 mg/ml-Janssen Animal Health Benelux) intramuscularly, in a dose of 1 ml/10 kg. At necropsy, all three joints were scored using a 0–4 scale, as described by van Grevenhof et al. [19] with 0 meaning OC-free, and 1–4 representing minimal, mild, moderate and severe lesions. The scoring was carried out independently by both researchers involved in the study.

### Histology

After necropsy, all joints were cut into 0.3 cm thick slices using a band saw, in a sagittal approach in order to visualise cartilage and subchondral bone (except for the right side samples of the pigs from the 2nd time point, which were cut in a frontal plane). Samples were directly processed for histology, except for the left side samples for the 3 pigs of the 3rd time point and the ten pigs from the last time point. Only few samples from these ten pigs were selected for logistical reasons, as it was technically too difficult to be able to analyse all samples. This selection was made using a simple visual examination of the cut tissue and first took place on the cut samples that showed OC lesions on radiographs during any one of the radiograph imaging time points. Two locations of that same joint (medial and lateral) were selected, to obtain an OC positive and negative sample (as normally only either the medial or lateral side is affected). If both sides were OC positive, then control samples were selected on the contralateral joint. Controls were randomized by selecting samples blindly from the list of samples which had corresponding negative radiographs for OC. From all the other radiographically negative joint locations random samples were chosen as control samples. All these pre-selected samples were examined by a veterinary clinician. All samples from this study were fixated in formalin, decalcified in 10 % EDTA and embedded in paraffin before being cut into 3 μm slices and stained with haematoxylin and eosin, and then scored on a 0–4 scale (Table 9).

**Table 9:** Schematic overview of the histological grading system used in this study

Grade	Classification	Signs
0	Normal	Vessels with erythrocytes, No necrotic areas, No irregularities on ossification front, Normal thickness of cartilage
1	Normal-Mod	Vessels with erythrocytes, Few to no very small necrotic areas, Some irregularities on ossification front, Some mild focal thickening of cartilage
2	Moderate	Vessels with/without erythrocytes, Some mild necrotic areas (not large), Mild irregularities on ossification front, Moderate focal thickening of cartilage
3	Mod-Severe	Vessels mostly without erythrocytes, Some large necrotic areas, Distinct irregularities on ossification front, areas of thickened cartilage
4	Severe	Vessels without or with extremely few erythrocytes, very large necrotic areas, Very severe irregularities of ossification front, Severe thickening of cartilage areas

## Statistical analysis

All statistical analyses were performed using the statistical program R (R: A Language and Environment for Statistical Computing, R Core Team, R Foundation for Statistical Computing, Vienna, Austria) and IBM SPSS Statistics 22 (IBM Corp. Released 2013. IBM SPSS Statistics for Windows, Version 22.0. Armonk, NY: IBM Corp).

### Bilaterally symmetrical aspect of OC

The degree of bilateral occurrence of presence and absence of lesions was examined by calculating the percentages of occurrences of total, partial or no bilateralism of histological findings between Left and Right joints.

### Radiography versus histology

The agreement strength between both methods was assessed using a receiver operating characteristic (ROC) curve that generates an area under the curve (AUC) statistic. This was performed on the histological and radiographic data from all examined joints from the last ten pigs of the study, where results for both techniques were available (Additional file 3).

### Visual scoring and pressure mat reproducibility

The frequencies of agreement and disagreement between both raters for all visual scoring aspects were counted and transformed into percentages (Table 4). For the pressure mat, an ICC (inter class correlation) was calculated using a one-way system, to measure the intra-assay variation of all four kinetic parameters from three successful runs each pig performed at all time points. The pressure mat software rating the subjects was considered as the constant and the pigs’ runs during one session as the random effects (Table 5). ICC outcomes were deduced using the Landis and Koch’s agreement scale [36].

### Visual scoring and pressure mat versus histology

Categorical H scores were transformed into continuous ones using the liability model used by van Grevenhof et al. for all joint locations [37, 38] to be able to compare them to the in-vivo method results which measure according to joint or animal level. Joint locations H scores were added up into joint level H scores and these were added up to make animal level H scores. A Spearman’s correlation test was used and a Benjamini and Hochberg false discovery rate was applied to *p* = 0.05 to determine significant p-values for individual tests. This study was approved by the Ethical committee of Utrecht University (DEC N. 2011.III.08.092.).

## Additional files

Additional file 1: Description: Illustration of the timeline of the analysis. (PDF 112 kb) Additional file 2: Description: Visual scoring characteristics and scoring categories for anatomical and mobility traits (PDF 86 kb) Additional file 3: Description: Final data set obtained for all pigs for histology and radiography Table 4a. Radiographic and histological joint assessments for the right side Table 4b. Radiographic and histological joint assessments for the left side. (PDF 186 kb)

## Abbreviations

ASI: asymmetry index
AUC: area under the curve
CA: contact area
Fmax: peak vertical force
FPD: flat panel detector
ICC: intra-class correlation
OC: osteochondrosis
Pmax: peak vertical pressure
PNR: point of no return
ROC: receiver operating characteristic
VI: vertical impulse
