# Supplementary material for: A longitudinal study on the performance of in vivo methods to determine the osteochondrotic status of young pigs
Source: BMC Vet Res. 2016 Mar 24;12:62. doi: 10.1186/s12917-016-0682-z (PMC4807589; doi:10.1186/s12917-016-0682-z)
Supplement: Additional file 1: — Description: Illustration of the timeline of the analysis. (PDF 112 kb) [file 12917_2016_682_MOESM1_ESM.pdf]

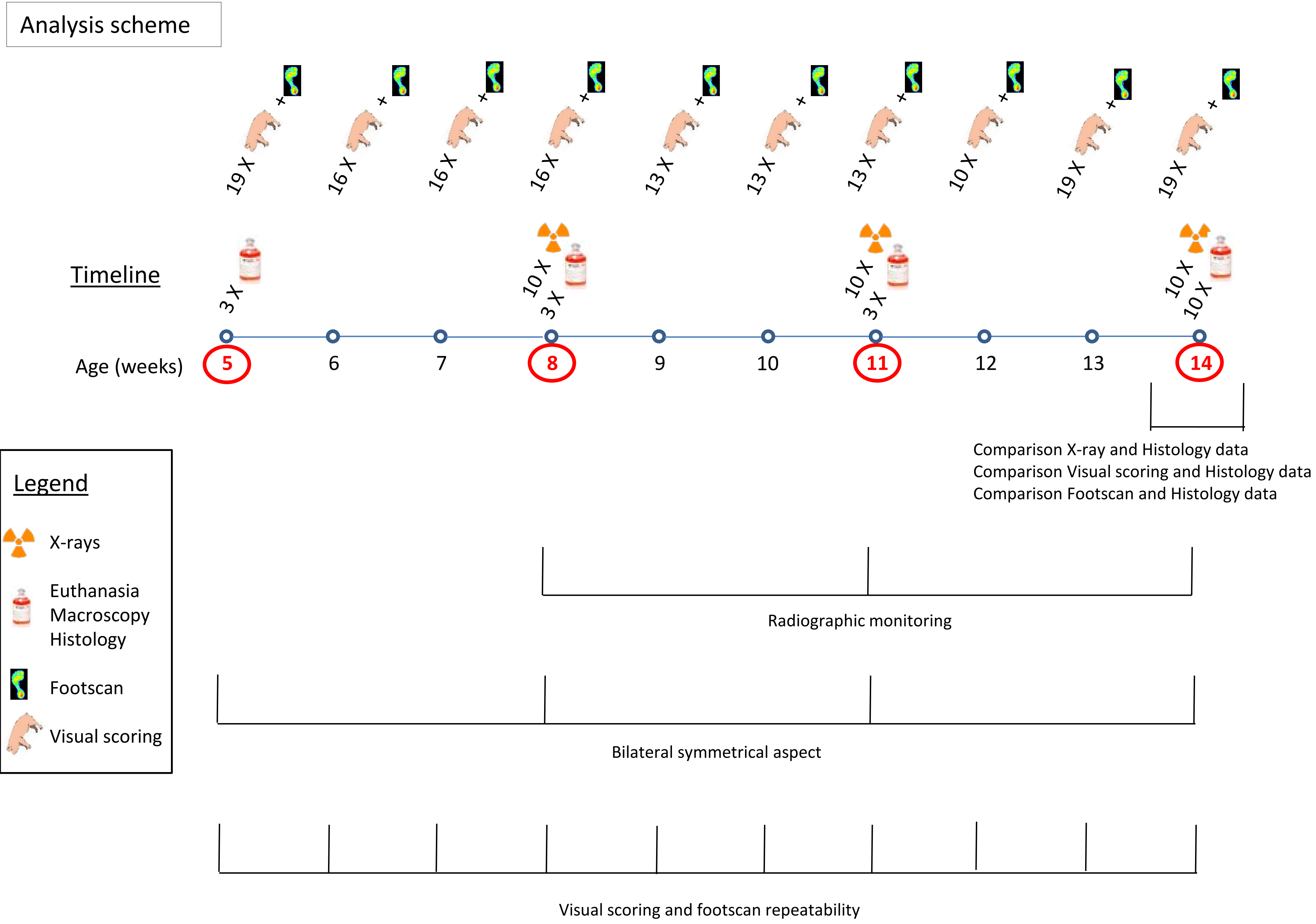
Analysis scheme
19 X
16 X
16 X
16 X
13 X
13 X
13 X
10 X
19 X
19 X
Timeline
3 X
10 X
3 X
10 X
3 X
10 X
10 X
Age (weeks)
5
6
7
8
9
10
11
12
13
14
Comparison X-ray and Histology data
Comparison Visual scoring and Histology data
Comparison Footscan and Histology data
Legend
X-rays
Euthanasia
Macroscopy
Histology
Footscan
Visual scoring
Radiographic monitoring
Bilateral symmetrical aspect
Visual scoring and footscan repeatability
