# Supplementary material for: A longitudinal study on the performance of in vivo methods to determine the osteochondrotic status of young pigs
Source: BMC Vet Res. 2016 Mar 24;12:62. doi: 10.1186/s12917-016-0682-z (PMC4807589; doi:10.1186/s12917-016-0682-z)
Supplement: Additional file 2: — Description: Visual scoring characteristics and scoring categories for anatomical and mobility traits (PDF 86 kb) [file 12917_2016_682_MOESM2_ESM.pdf]

Additional File 2. Visual scoring characteristics and scoring categories for anatomical and mobility traits

Visual characteristics		Scoring categories		
Anatomical traits		“Below 4” (1-4)	“Normal” (4-6)	“Above 6” (6-9)
Fore legs	O or X shape	O shape		X shape
	Sickled or Buckled	Sickled		Buckled
	Steep or low angled pasterns	Steep		Low
	Claw size	Inner claw smaller		Inner claw larger
Hind legs	O or X shape	O shape		X shape
	Straight or Sickled	Straight		Sickled
	Steep or low angled pasterns	Steep		Low
	Claw size	Inner claw smaller		Inner claw larger
Hams	Width	Thin		Large
Mobility traits		Below 9 (1-9)		Above 9 (9-10)
	Gait pattern	Laboured to severely laboured		Smooth
		Below 2 (1-2)	Above 2 (2-10)	
	Twisting hocks	No twisting	Noticeable twisting to Severe twisting	
	Swaying hind	No swaying	Noticeable Swaying Severe swaying	
