# Supplementary material for: A longitudinal study on the performance of in vivo methods to determine the osteochondrotic status of young pigs
Source: BMC Vet Res. 2016 Mar 24;12:62. doi: 10.1186/s12917-016-0682-z (PMC4807589; doi:10.1186/s12917-016-0682-z)
Supplement: Additional file 3: — Description: Final data set obtained for all pigs for histology and radiography Table 4a. Radiographic and histological joint assessments for the right side Table 4b. Radiographic and histological joint assessments for the left side. (PDF 186 kb) [file 12917_2016_682_MOESM3_ESM.pdf]

Additional file 4. Final data set obtained for all pigs for histology and radiography

Table 4a. Radiographic and histological joint assessments for the right side

			Humerus		Ulna		Femur		Tibia superior		Tibia Inferior		Talus	
Pig ID	Gender	Date	Medial_R	Lateral_R	Medial_R	Lateral_R	Medial_R	Lateral_R	Medial_R	Lateral_R	Medial_R	Lateral_R	Medial_R	Lateral_R
2571	Male	5	H+R	H+R	H+R	H+R	H+R	H+R	R	R	H+R	H+R	H+R	H+R
2372	Female	5	H+R	H+R	H+R	H+R	H+R	H+R	H+R	H+R	H+R	H+R	H+R	H+R
2381	Male	5	H+R	H+R	H+R	H+R	H+R	H+R	H+R	H+R	H+R	H+R	H+R	H+R
2387	Female	8	H+R	H+R	H+R	H+R	H+R	H+R	H+R	H+R	H+R	H+R	H+R	H+R
2405	Female	8	H+R	H+R	H+R	H+R	H+R	H+R	H+R	H+R	H+R	H+R	H+R	H+R
2585	Male	8	H+R	H+R	H+R	H+R	H+R	H+R	H+R	H+R	H+R	H+R	H+R	H+R
1932	Female	11	H+R	H+R	H+R	H+R	H+R	H+R	H+R	H+R	H+R	H+R	H+R	H+R
2412	Male	11	H+R	H+R	H+R	H+R	H+R	H+R	H+R	H+R	H+R	H+R	H+R	H+R
2566	Female	11	H+R	H+R	H+R	H+R	H+R	H+R	H+R	H+R	H+R	H+R	H+R	H+R
2420	Female	14	H+R	R	R	H+R	H+R	H+R	R	R	H+R	H+R	H+R	R
2431	Male	14	H+R	H+R	R	H+R	H+R	H+R	H+R	R	H+R	R	H+R	R
2441	Male	14	H+R	H+R	R	R	H+R	H+R	R	R	H+R	R	H+R	R
2454	Female	14	H+R	R	R	H+R	H+R	H+R	R	R	H+R	R	H+R	R
2461	Male	14	R	H+R	R	R	H+R	H+R	H+R	R	H+R	R	H+R	R
2583	Female	14	H+R	H+R	R	H+R	H+R	H+R	R	R	H+R	R	H+R	R
2593	Female	14	H+R	R	R	H+R	H+R	H+R	R	R	H+R	R	H+R	H+R
2599	Male	14	H+R	R	R	H+R	H+R	H+R	H+R	R	H+R	R	H+R	H+R
2396	Male	14	H+R	H+R	R	H+R	H+R	H+R	R	R	H+R	R	H+R	R
1939	Male	14	H+R	R	R	H+R	H+R	R	R	R	H+R	R	R	H+R

Table 4b. Radiographic and histological joint assessments for the left side

			Humerus		Ulna		Femur		Tibia superior		Tibia Inferior		Talus	
Pig ID	Gender	Date	Medial_L	Lateral_L	Medial_L	Lateral_L	Medial_L	Lateral_L	Medial_L	Lateral_L	Medial_L	Lateral_L	Medial_L	Lateral_L
2571	Male	5	H+R	H+R	H+R	H+R	H+R	H+R	H+R	H+R	H+R	H+R	H+R	H+R
2372	Female	5	H+R	H+R	H+R	H+R	H+R	H+R	H+R	H+R	H+R	H+R	H+R	H+R
2381	Male	5	H+R	H+R	H+R	H+R	H+R	H+R	R	R	R	R	H+R	H+R
2387	Female	8	H+R	H+R	H+R	H+R	H+R	H+R	H+R	H+R	H+R	H+R	H+R	H+R
2405	Female	8	H+R	H+R	H+R	H+R	H+R	H+R	H+R	H+R	H+R	H+R	H+R	H+R
2585	Male	8	H+R	H+R	H+R	H+R	H+R	H+R	H+R	H+R	H+R	H+R	H+R	H+R
1932	Female	11	R	R	R	R	R	R	R	R	R	R	R	R
2412	Male	11	R	R	R	R	R	R	R	R	R	R	R	R
2566	Female	11	R	R	R	R	R	R	R	R	R	R	R	R
2420	Female	14	H+R	H+R	R	R	H+R	H+R	H+R	R	R	R	R	H+R
2431	Male	14	H+R	H+R	R	R	H+R	H+R	R	R	R	R	R	H+R
2441	Male	14	H+R	R	R	R	H+R	H+R	R	R	R	R	R	H+R
2454	Female	14	H+R	H+R	R	R	H+R	H+R	R	R	R	R	R	H+R
2461	Male	14	H+R	H+R	R	R	H+R	H+R	R	R	R	R	R	H+R
2583	Female	14	H+R	R	R	R	H+R	H+R	R	R	R	R	R	H+R
2593	Female	14	R	H+R	R	R	H+R	H+R	R	H+R	R	R	H+R	R
2599	Male	14	R	H+R	R	R	H+R	H+R	R	R	R	R	R	H+R
2396	Male	14	R	H+R	R	R	H+R	H+R	R	R	R	R	R	H+R
1939	Male	14	R	H+R	R	R	H+R	H+R	R	R	R	R	H+R	H+R

Legend

H: Histological assessments
R: Radiological assessments
Blue: OC radiographically negative samples (control samples)
Red: OC radiographically positive samples (at one or multiple time points) found to be positive during visual examination of cut sample prior to histology
Brown: OC radiographically positive sample (at one time point) that was found to be negative during visual examination of cut sample prior to histology
Purple: OC radiographically positive sample, unsuccessfully processed for histology
